# Native cyclase-associated protein and actin from *Xenopus laevis* oocytes form a unique 4:4 complex with a tripartite structure

**DOI:** 10.1016/j.jbc.2021.100649

**Published:** 2021-04-09

**Authors:** Noriyuki Kodera, Hiroshi Abe, Phuong Doan N. Nguyen, Shoichiro Ono

**Affiliations:** 1 WPI-Nano Life Science Institute, Kanazawa University, Kanazawa, Ishikawa, Japan; 2 Department of Biology, Graduate School of Science, Chiba University, Chiba, Japan; 3 Departments of Pathology and Cell Biology, Emory University School of Medicine, Atlanta, Georgia, USA; 4 Winship Cancer Institute, Emory University School of Medicine, Atlanta, Georgia, USA

**Keywords:** actin, cyclase-associated protein, atomic force microscopy (AFM), cytoskeleton, *Xenopus*, protein complex, APTES, (3-aminopropyl)triethoxysilane, Arm-HS, arm in a high state, Arm-LS, arm in a low state, CAP, cyclase-associated protein, CARP, CAP and X-linked retinitis pigmentosa 2 protein, HFD, helical folded domain, HS-AFM, high-speed atomic force microscopy, INF2, inverted formin 2, MGD, middle globular domain, SEC-MALS, size-exclusion chromatography–multiangle light scattering, WH2, Wiskott Aldrich Syndrome Protein homology 2, XAC, *Xenopus* actin depolymerizing factor/cofilin, XCAP1, *Xenopus* cyclase-associated protein 1

## Abstract

Cyclase-associated protein (CAP) is a conserved actin-binding protein that regulates multiple aspects of actin dynamics, including polymerization, depolymerization, filament severing, and nucleotide exchange. CAP has been isolated from different cells and tissues in an equimolar complex with actin, and previous studies have shown that a CAP–actin complex contains six molecules each of CAP and actin. Intriguingly, here, we successfully isolated a complex of *Xenopus* cyclase-associated protein 1 (XCAP1) with actin from oocyte extracts, which contained only four molecules each of XCAP1 and actin. This XCAP1–actin complex remained stable as a single population of 340 kDa species during hydrodynamic analyses using gel filtration or analytical ultracentrifugation. Examination of the XCAP1–actin complex by high-speed atomic force microscopy revealed a tripartite structure: one middle globular domain and two globular arms. The two arms were observed in high and low states. The arms at the high state were spontaneously converted to the low state by dissociation of actin from the complex. However, when extra G-actin was added, the arms at the low state were converted to the high state. Based on the known structures of the N-terminal helical-folded domain and C-terminal CARP domain, we hypothesize that the middle globular domain corresponds to a tetramer of the N-terminal helical-folded domain of XCAP1 and that each arm in the high state corresponds to a heterotetramer containing a dimer of the C-terminal CARP domain of XCAP1 and two G-actin molecules. This novel configuration of a CAP–actin complex should help to understand how CAP promotes actin filament disassembly.

Regulated assembly and disassembly of actin filaments are vital to the diverse function of the actin cytoskeleton (1). Cyclase-associated protein (CAP) is one of the actin-regulatory proteins that control multiple key aspects of actin filament dynamics (2, 3). CAP was originally identified in yeast as a protein that binds to adenylyl cyclase and is involved in the Ras signaling pathway (4, 5). However, CAP was later recognized as an actin-binding protein in a variety of eukaryotes. CAP binds to actin monomers and inhibits polymerization (6). CAP also promotes exchange of actin-bound nucleotides in competition with cofilin and increases ATP-bound actin monomers that are readily available for polymerization (7, 8, 9). In addition, CAP and cofilin interact with actin filaments to enhance severing (10, 11) and monomer dissociation from the pointed ends (12, 13). A combination of CAP and twinfilin also enhances actin monomer dissociation from filament ends (14, 15). CAP is involved in a number of cellular events that require actin remodeling in various cell types and tissues. For example, CAP is essential for muscle sarcomere organization in *Caenorhabditis elegans* (16) and mice (17), and deficiency of CAP2, a mammalian CAP isoform, causes cardiomyopathy in mice (18, 19) and humans (20).

Intriguingly, when nonrecombinant native CAP is isolated from tissues or cells, actin is associated with CAP in a multimeric complex at an equimolar ratio and cannot be dissociated without harsh conditions. Porcine CAP (originally reported as ASP-56) was isolated from platelets as a complex with actin, and actin was finally dissociated from CAP by 3 M urea (21). Similar CAP–actin complex has been isolated from yeast (8), bovine thymus (11), and mouse brain (22). The CAP–actin complex promotes actin filament disassembly in the presence of cofilin (8, 11). In addition, recent studies have shown that the CAP–actin complex containing acetylated actin is an inhibitor of inverted formin 2 (INF2) (22, 23). Thus, the CAP and actin have biological functions as a complex, but how the complex is assembled and why the complex formation is important for its functions remain unknown.

The native complex of yeast CAP (also known as Srv2) and actin is a 6:6 complex of ∼600 kDa (8), which can be reconstituted from purified components (24). The CAP–actin complex from mouse brain is also in a similar size (22). The N-terminal half of yeast and mouse CAPs form a hexameric “*shuriken*” structure, which is mediated by oligomerization of a putative coiled-coil region at the most N-terminus (10, 25) and dimerization of the helical-folded domain (HFD) (12, 26, 27). The C-terminal half of CAP contains a CAP and X-linked retinitis pigmentosa 2 protein (CARP) domain that dimerizes through the most C-terminal dimerization motif (28, 29). The CARP domain of CAP binds to actin monomer (6, 30, 31, 32), and a CARP dimer and two actin molecules form a compact globular structure (33). Although we know structures of parts of the CAP–actin complex, we still have limited knowledge on the structure of the entire complex. Furthermore, a recent study has demonstrated that the N-terminal regions of human CAP1 and CAP2 primarily form tetramers instead of hexamers (34). Therefore, whether the 6:6 configuration is conserved among CAP–actin complexes from different sources remains unknown. In this study, we purified a complex of *Xenopus* CAP1 and actin and demonstrated that the complex contained the two proteins in a 4:4 stoichiometric ratio, which is a novel configuration of the CAP–actin complex.

## Results

### *Xenopus* CAP1 (XCAP1) and actin form a 4:4 complex

**Figure 1:**
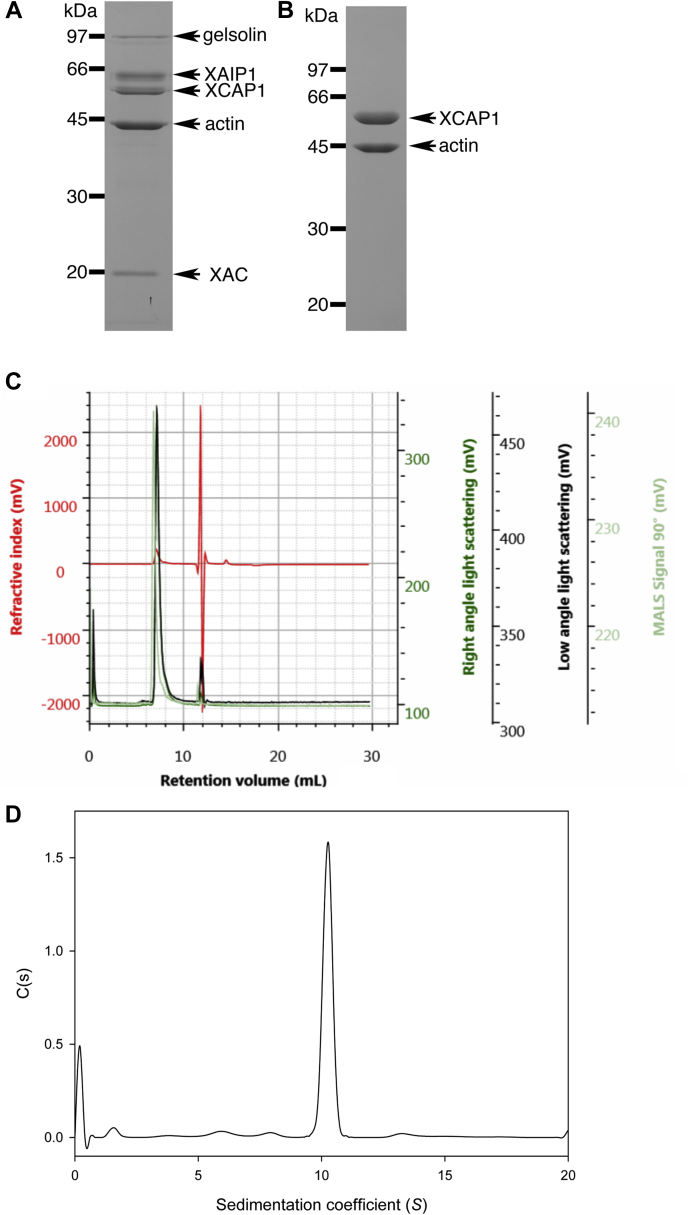
**Determination of native molecular weight of the XCAP1–actin complex.***A* and *B*, purification of XCAP1–actin complex from *Xenopus* oocyte extracts. *A*, proteins that bound to the XAC-affinity column were eluted, separated by SDS-PAGE, and stained with Coomassie *Brilliant Blue*. Each band was identified by peptide sequencing as shown on the *right* of the gel. *B*, a complex of XCAP1 and actin was isolated after anion-exchange chromatography and hydroxyapatite chromatography. Positions of molecular mass markers in kDa are shown on the *left*. *C*, SEC-MALS analysis of the XCAP1–actin complex. Purified XCAP1–actin complex was applied to size-exclusion chromatography, and refractive index (mV, *red*), right-angle light scattering (mV, *dark green*), low-angle light scattering (mV, *black*), MALS signal at 90° (mV, *light green*) were monitored. *D*, analytical ultracentrifugation analysis of the XCAP1–actin complex. A single peak of 10S was detected indicating that the XCAP1–actin complex was stable.

We purified a native complex of CAP and actin from *Xenopus* oocyte extracts (Fig. 1). When *Xenopus* oocyte extracts were applied to a column in which glutathione S-transferase (GST)-fused *Xenopus* ADF/cofilin (XAC) was immobilized, several proteins specifically bound to the column as described previously (35) (Fig. 1*A*). We reported that the 65-kDa, 42-kDa, and 19-kDa proteins were *Xenopus* actin-interacting protein 1 (XAIP1), actin, and XAC, respectively (35). Peptide sequencing identified that the 94-kDa and 60-kDa proteins were gelsolin (36) and cyclase-associated protein 1 (XCAP1) (37), respectively. We attempted to isolate XCAP1 using anion-exchange chromatography followed by hydroxyapatite chromatography, but XCAP1 and actin were not separated during these procedures and were instead purified together in an equimolar ratio (Fig. 1*B*). Further gel filtration chromatography using Sephadex G-200 also resulted in coelution of XCAP1 and actin in a single peak at ∼390 kDa (our unpublished observation), which is much larger than XCAP1 or actin alone, or a 1:1 complex, indicating that they form a stable multimeric complex.

Native molecular mass of the XCAP1–actin complex was determined more accurately by two different methods: size-exclusion chromatography coupled with multiangle light scattering (SEC-MALS) and analytical ultracentrifugation. In SEC-MALS, the XCAP1–actin complex was resolved as a single peak with a molecular mass of 340 kDa (Fig. 1*C*). There were no detectable peaks that corresponded to dissociated XCAP1 or actin, indicating that the XCAP1–actin complex was stable during the SEC-MALS analysis. Likewise, in analytical ultracentrifugation, the XCAP1–actin complex was resolved as a single peak of 337 kDa (*S* = 10) (Fig. 1*D*), which agrees with the result of SEC-MALS. Considering the molecular masses of individual XCAP1 (52 kDa) and actin (42 kDa), the native molecular mass of the XCAP1–actin complex most closely matched with that of a 4:4 complex (calculated molecular mass of 376 kDa). The experimentally determined molecular mass was ∼10% smaller than the calculated molecular mass. This could be due to partial dissociation of the complex during the analyses, which is a known limitation in some cases of SEC-MALS experiments (38). Since CAPs are known to bind to actin monomers, the XCAP1–actin complex most likely contains G-actin. Therefore, these results indicate that the native XCAP1 and G-actin form a stable complex at a 4:4 stoichiometric ratio.

### XCAP1–actin complex has a tripartite structure as revealed by high-speed atomic force microscopy

**Figure 2:**
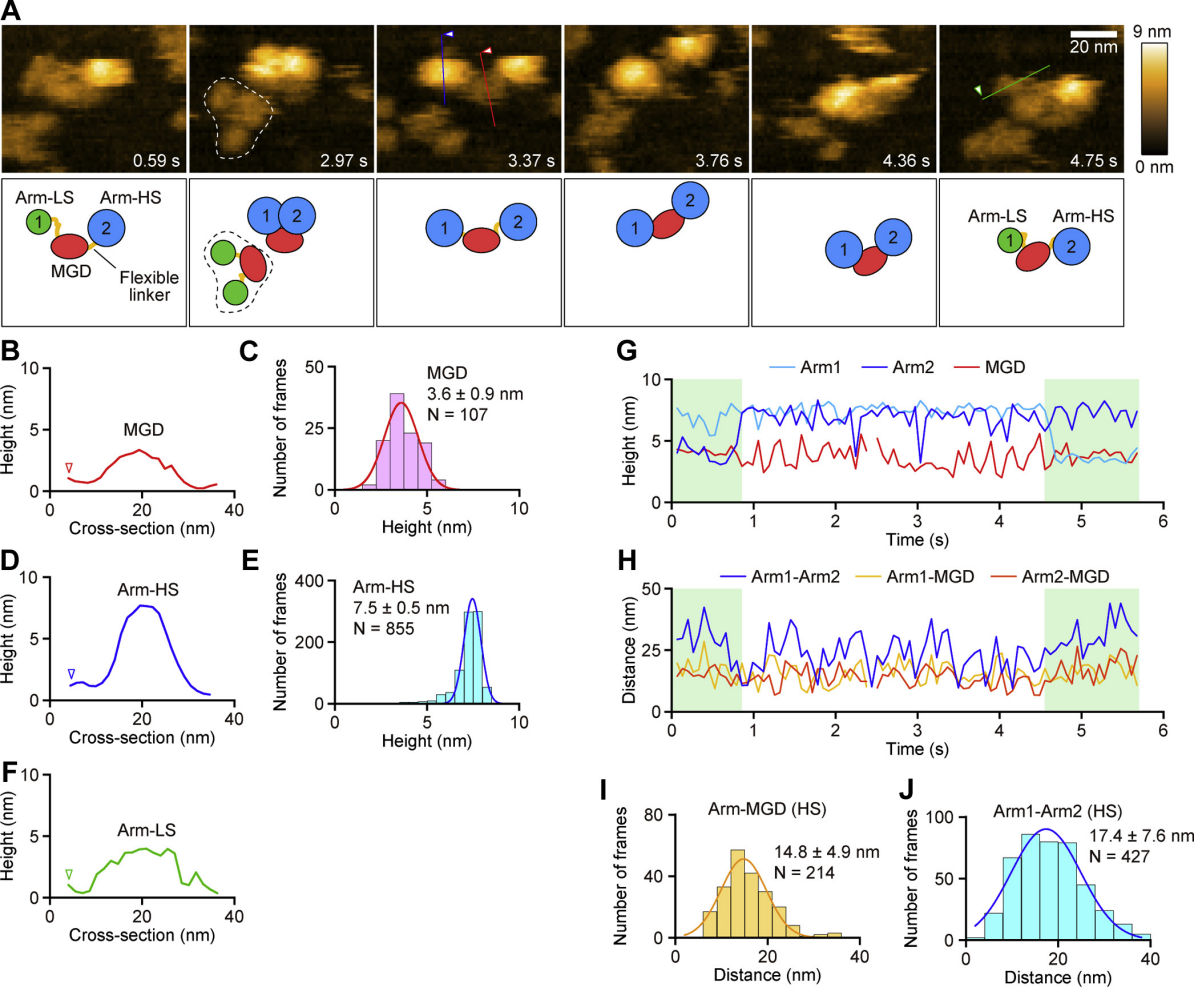
**High-speed atomic force microscopy reveals a tripartite structure of the XCAP1–actin complex.***A*, time-lapse HS-AFM images of the XCAP1–actin complex on a mica surface (see Supplementary Movie S1). Scanning area was 80 × 64 nm^2^ with 64 × 48 pixels. Imaging rate was 66 ms/frame (∼15 fps). Bar, 20 nm. Schematic representation of molecular features is shown in the *bottom panels*: middle globular domain (MGD, *red*), arm in the low state (Arm-LS, *green*), and arm in the high state (Arm-HS, *blue*). The complex indicated by *dashed lines* in the second frame had both arms in Arm-LS throughout the observation (see Fig. 3 for quantitative analysis of Arm-LS). *B*–*F*, cross-sectional analyses of MGD (*B*, *red*), Arm-HS (*D*, *blue*), and Arm-LS (*F*, *green*) at the *straight colored lines* drawn on the images in *A*. Height distributions of MGD (*C*) and Arm-HS (*E*) and single Gaussian fitting yielded average heights of MGD and Arm-HS as indicated in the figure. *G*, time course of the heights of three globular domains. *Green-shaded areas* indicate periods when one of the arms was in the low state. *H*–*J*, time course of the distances between the domains at their highest points (*H*). Distribution of the distance between MGD and one of the arms (*I*) and between two arms (*J*) and single Gaussian fitting yielded average distances as indicated in the figure. Arm-HS was selected in these analyses.

**Figure 3:**
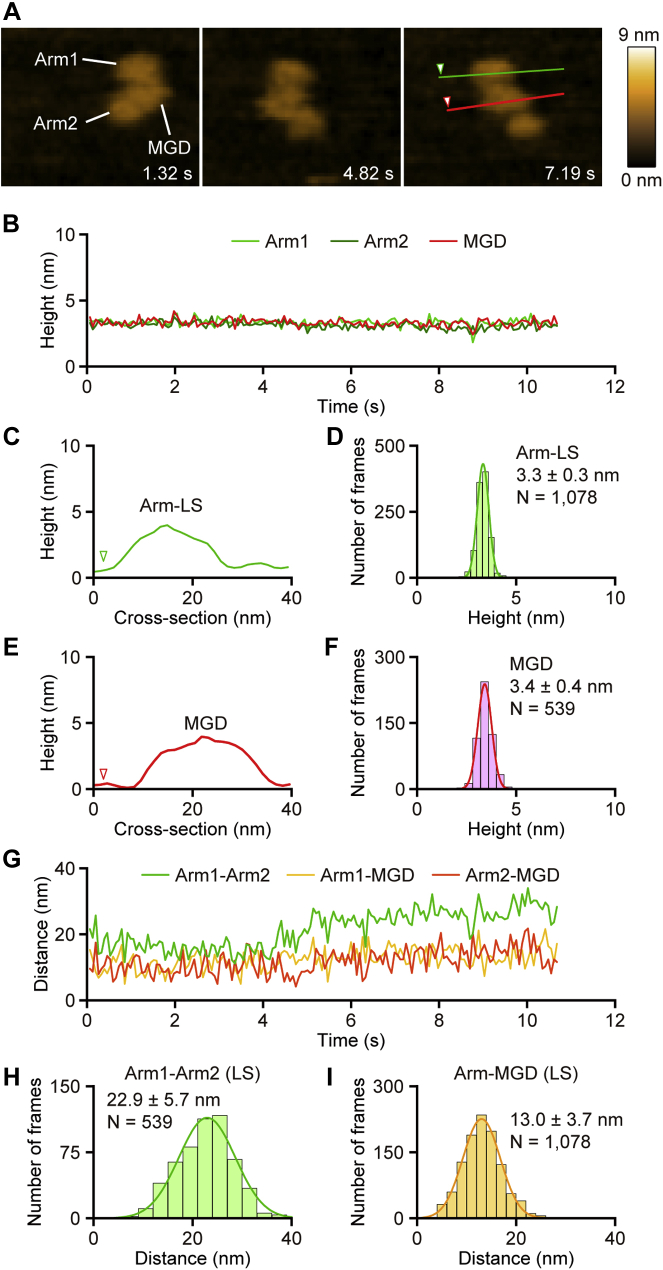
**The XCAP1–actin complex with both arms in a low state is stable.***A*, time-lapse HS-AFM images of the XCAP1–actin complex containing both arms in Arm-LS on a mica surface (see Supplementary Movie S2). Scanning area was 80 × 64 nm^2^ with 64 × 48 pixels. Imaging rate was 66 ms/frame (∼15 fps). Bar, 20 nm. *B*, time course of the heights of three globular domains. *C*–*F*, cross-sectional analyses of Arm-LS (*C*, *green*) and MGD (*E*, *red*) at the *straight colored lines* drawn on the image in *A*. Height distributions of Arm-LS (*D*) and MGD (*F*) and single Gaussian fitting yielded average heights of Arm-LS and MGD as indicated in the figure. *G*–*I*, time course of the distances between the domains at their highest points (*G*). Distribution of the distance between two arms (*H*) and between MGD and one of the arms (*I*) and single Gaussian fitting yielded average distances as indicated in the figure.

Structure of the XCAP1–actin complex in its native state was examined by high-speed atomic force microscopy (HS-AFM) (Figs. 2 and 3). Typical images on mica surfaces showed that the complex consisted of three globular domains (Fig. 2*A*), which we designated as the middle globular domain (MGD, shown in red in Fig. 2*A* cartoons) and two arms (Arm 1 and Arm 2, shown in green or blue in Fig. 2*A* cartoons). The height of MGD was 3.6 ± 0.9 nm (n = 107) (Fig. 2, *B* and *C*) and remained relatively stable during time-lapse imaging (Fig. 2, *A* and *G*, Supplementary Movie S1). By contrast, the two arms were observed in two different states: a high state (Arm-HS, shown in blue in Fig. 2*A* cartoons) and a low state (Arm-LS, shown in green in Fig. 2*A* cartoons, also see Fig. 3). The height of Arm-HS was 7.5 ± 0.5 nm (n = 855) (Fig. 2, *D* and *E*), while that of Arm-LS was 3.3 ± 0.3 nm (n = 1078) (Figs. 2*F* and 3, *C* and *D*). In some cases, the arms transitioned either from Arm-LS to Arm-HS (Fig. 2, *A* and *G*, blue line at ∼0.9 s) or from Arm-HS to Arm-LS (Fig. 2, *A* and *G*, green line at 4.5 s), suggesting that association or dissociation of a component, presumably G-actin, occurred during observations. Over the periods of HS-AFM observations, Arm-HS gradually decreased, while Arm-LS predominated, suggesting that Arm-HS was converted to Arm-LS by dissociation of actin over time likely due to repeated tapping by the AFM probe and adsorption of the complex on the surface (see below). Some of the complexes had Arm-LS throughout the observations (Fig. 2*A*, indicated by dashed line, Fig. 3*A*, Supplementary Movie S2), and the height of Arm-LS was 3.3 ± 0.3 nm (n = 1078) (Fig. 3, *B*–*D*).

The two arms were very dynamic when they were in Arm-HS (Fig. 2*A*, Supplementary Movie S1), but restricted within 14.8 ± 4.9 nm (n = 214) of MGD (Fig. 2, *H* and *I*) as if the arms were connected to MGD by flexible linkers. The distance between the highest points of the two arms in Arm-HS fluctuated in a wide range with an average of 17.4 ± 7.6 nm (n = 427) (Fig. 2, *H* and *J*), further supporting the presence of flexible linkers between MGD and each arm. However, once arms were converted from Arm-HS to Arm-LS, they were stabilized in Arm-LS (Fig. 3, *A*–*D*, Supplementary Movie S2), while MGD remained unchanged (Fig. 3, *B*, *E* and *F*). The distance between two arms became wider [22.9 ± 5.7 nm (n = 539)], whereas that between each arm and MGD became narrower [13.0 ± 3.7 nm (n = 1078)]. These results suggest that Arm-LS was physically stabilized by adsorption to the mica surface.

**Figure 4:**
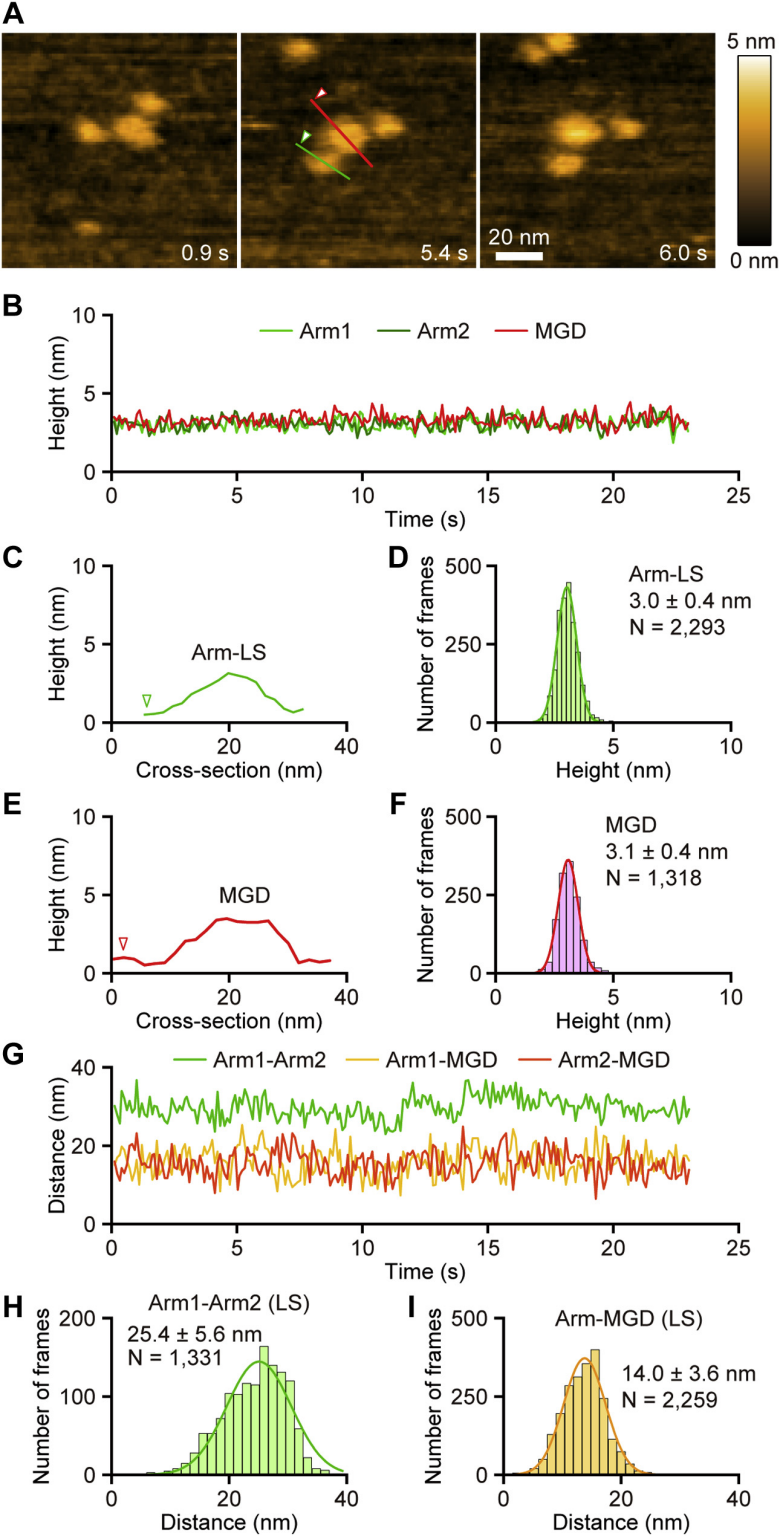
**Strong adsorption of the XCAP1–actin complex to a charged surface stabilizes two arms in a low state.***A*, time-lapse HS-AFM images of the XCAP1–actin complex on an APTES-treated mica surface (see Supplementary Movie S3). Scanning area was 100 × 100 nm^2^ with 80 × 80 pixels. Imaging rate was 100 ms/frame (10 fps). Bar, 20 nm. *B*, time course of the heights of three globular domains. *C*–*F*, cross-sectional analyses of Arm-LS (*C*, *red*) and Arm-LS (*E*, *green*) at the *straight colored lines* drawn on the image in *A*. Height distributions of Arm-LS (*D*) and MGD (*F*) and single Gaussian fitting yielded average heights of MGD and Arm-HS as indicated in the figure. *G*–*I*, time course of the distances between the domains at their highest points (*G*). Distribution of the distance between two arms (*H*) and between MGD and one of the arms (*I*) and single Gaussian fitting yielded average distances as indicated in the figure.

To test how surface adsorption affects the molecular features of the XCAP1–actin complex, we used a mica surface that was treated with APTES, which adds positive charges to the surface and causes nonspecific strong adsorption of proteins. On APTES-treated mica, the two arms were almost always detected in the low state (Arm-LS) with the height of 3.0 ± 0.4 nm (n = 2293) (Fig. 4, *A*–*D*, Supplementary Movie S3). The height and shape of MGD were indistinguishable between normal and APTES-treated mica surfaces (Fig. 4, *A*, *B*, *E*, and *F*). The distance between the two arms remained relatively constant at 25.4 ± 5.6 nm (n = 1331) (Fig. 4, *G* and *H*), which is much wider than that of two arms in Arm-HS on normal mica surfaces (Fig. 2*I*), suggesting that the two arms were strongly immobilized on the surface and spread apart. By contrast, the distance between MGD and an arm remained constant on the APTES-treated surface (Fig. 4, *G* and *I*) in a similar manner to MGD and Arm-LS on the normal surface (Fig. 3*I*). These observations suggest that strong adsorption of the XCAP1–actin complex onto a solid surface artificially converts Arm-HS to Arm-LS by causing dissociation of an arm-bound component, which we hypothesize to be G-actin.

**Figure 5:**
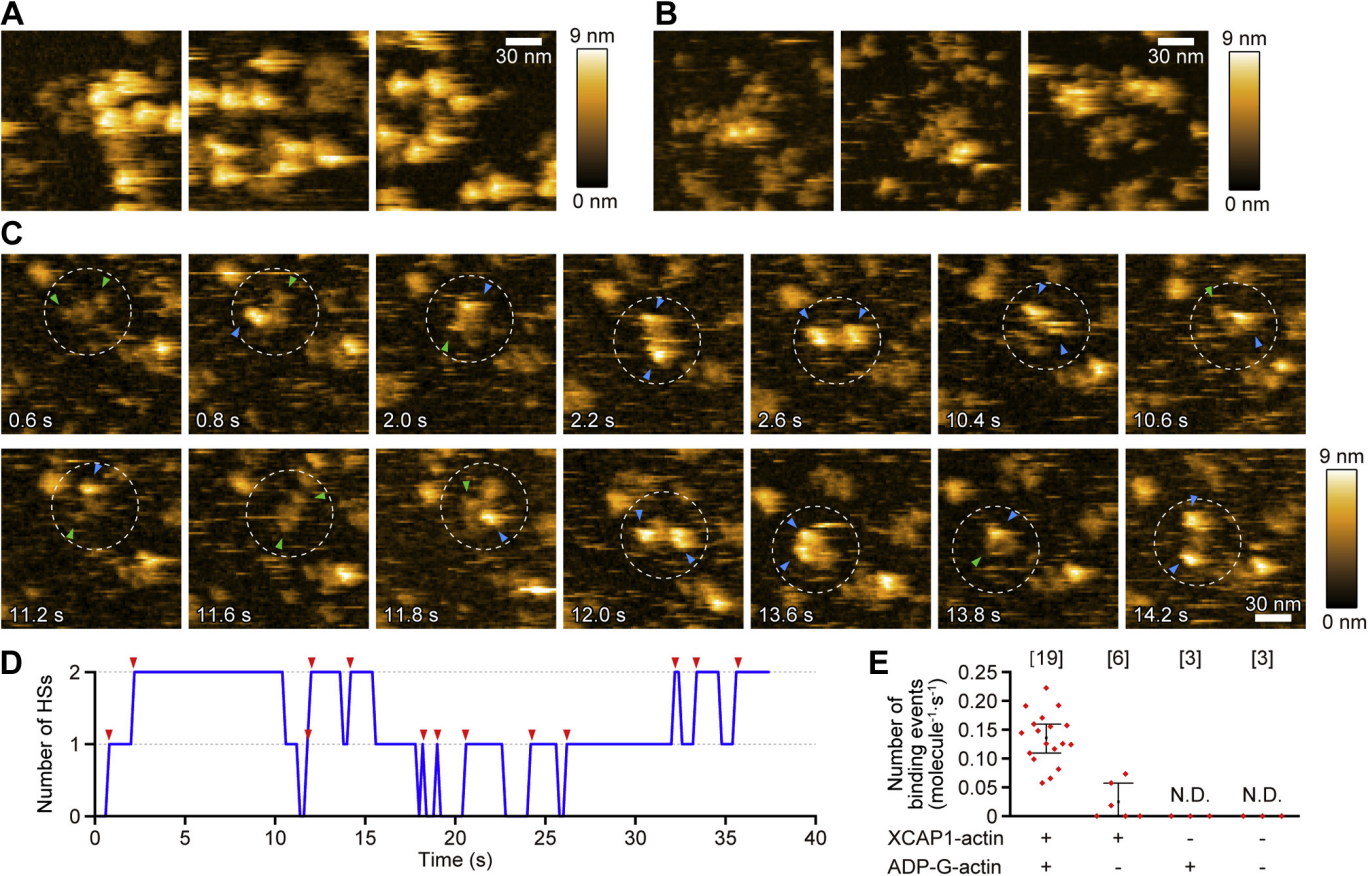
**Addition of ADP-G-actin to the XCAP1–actin complex promotes conversion of arms from a low to high state.***A* and *B*, representative images of the XCAP1–actin complex on a mica surface immediately after observation (*A*) and after 15 min of observation (*B*). *C*–*E*, time-lapse HS-AFM images of the XCAP1–actin complex after addition of final 100 nM ADP-G-actin (see Supplementary Movie S4). The complex indicated by a dashed circle showed frequent and reversible conversions between Arm-LS (*green arrowheads*) and Arm-HS (*blue arrowheads*) as plotted in (*D*). *Red arrowheads* in (*D*) indicate conversion events from Arm-LS to Arm-HS. *E*, quantitative analysis of frequency of binding events (conversion from Arm-LS to Arm-HS; molecules^−1^ s^−1^). In the presence of ADP-G-actin only and in the absence of the XCAP1–actin complex and ADP-G-actin, no objects that matched the size of Arm-LS or Arm-HS were observed, (N. D., none detected). Sample numbers are shown in the *brackets*.

To test whether the height conversion of the arms is due to G-actin dissociation and association, we examined effects of additional free ADP-G-actin on the XCAP1–actin complex (Fig. 5). Freshly prepared samples of the XCAP1–actin complex contained mostly Arm-HS (Fig. 5*A*). However, after 15 min, most of them were converted to Arm-LS (Fig. 5*B*). After most of the arms were converted to Arm-LS (after ∼20 min), final 100 nM ADP-G-actin was added, and molecular features of the complex were observed over time (Fig. 5, *C* and *D*). In the presence of ADP-G-actin, frequent and reversible conversions of the arms between Arm-LS and Arm-HS were observed (Fig. 5*C*, Supplementary Movie S4). In the example shown in Figure 5*C* (dashed circle), initially both arms were Arm-LS (green arrowheads) at 0.6 s (Fig. 5*C*, top left panel), one arm was then converted to Arm-HS (blue arrowhead) at 0.8 s (Fig. 5*C*, top second panel), and another arm was converted to Arm-HS at 2.2 s (Fig. 5*C*, top fourth panel). Conversely, conversions from Arm-HS to Arm-LS were also observed (Fig. 5*C*, top right panel at 10.6 s and bottom second panel at 11.6 s). A plot of conversion events over time (Fig. 5*D*) indicates that the conversion between Arm-LS and Arm-HS was reversible and independently occurred at each arm without coordination between the two arms within a single complex. The rate of conversion events from Arm-LS to Arm-HS in the presence of ADP-G-actin was 0.14 ± 0.04 molecules^−1^ s^−1^ (Fig. 5*E*). Even in the absence of ADP-G-actin, conversion of Arm-LS to Arm-HS was occasionally observed at a rate of 0.03 ± 0.03 molecules^−1^ s^−1^ (Fig. 5*E*) most likely due to rebinding of dissociated G-actin to the complex, which is consistent with the spontaneous transition from Arm-LS to Arm-HS as demonstrated in Figure 1*A*. In the presence of ADP-G-actin alone or in the absence of the XCAP1–actin complex and ADP-G-actin, no objects that matched the size of Arm-LS or Arm-HS were observed (N.D.: none detected, Fig. 5*E*). These results strongly suggest that G-actin is a component of the two arm domains of the XCAP1–actin complex.

## Discussion

**Figure 6:**
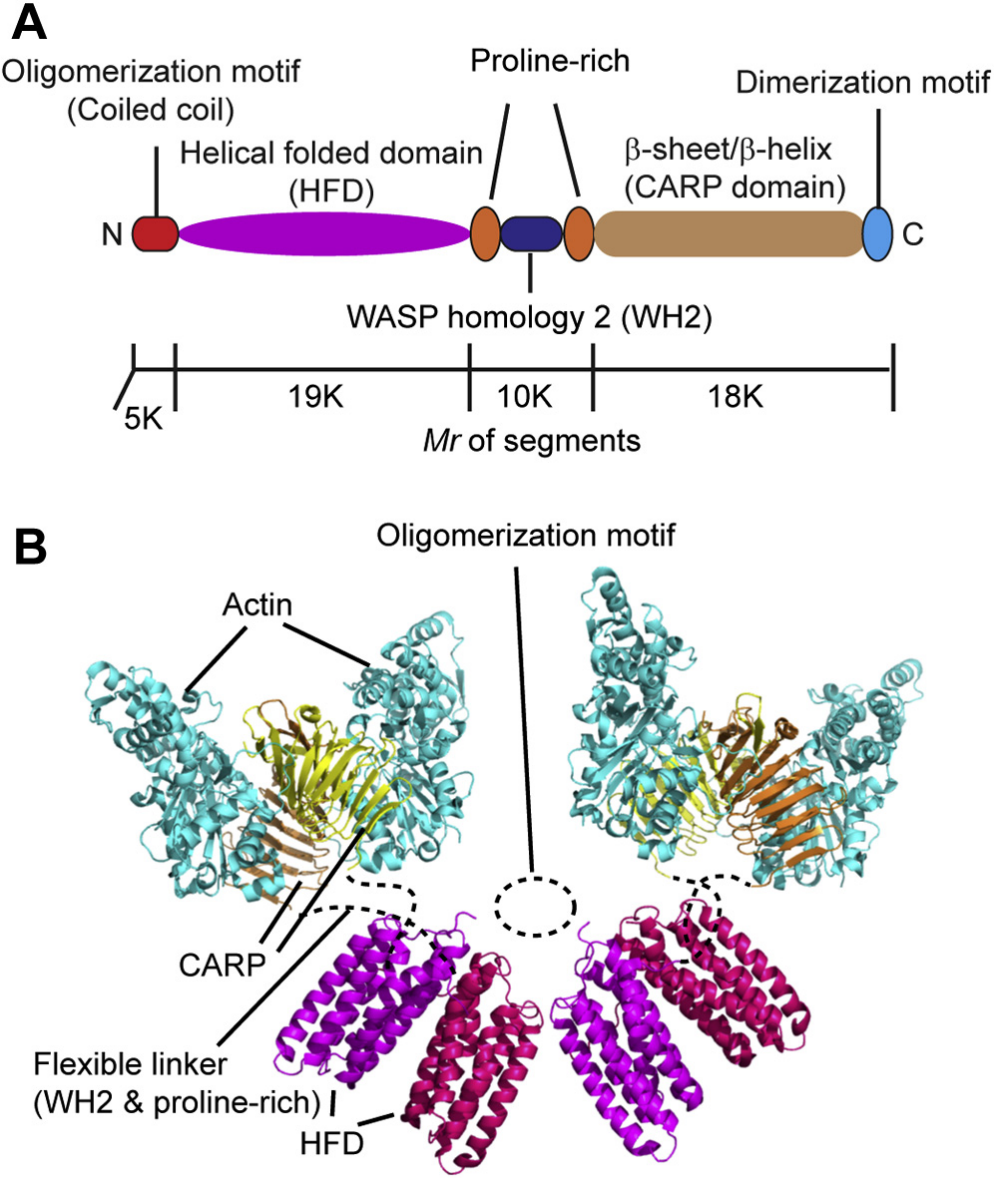
**Model of the CAP–actin complex.***A*, domain structure of CAP [adopted from (2)]. Approximate molecular masses of segments are shown. *B*, a model of the CAP–actin complex. Crystal structures of HFD of mouse CAP1 (Protein Data Bank accession ID: 6RSQ) and CARP domain of mouse CAP1 bound to actin (Protein Data Bank accession ID: 6FM2) were used to reconstruct a CAP–actin complex at a 4:4 stoichiometric ratio. Putative locations of oligomerization motif and flexible linkers are indicated by *dashed lines*.

Based on known biochemical and biophysical properties of CAP from other species, we propose a model for the structure of the XCAP1–actin complex, which is in the appearance of two “butterflies” (CARP/G-actin) and a “flower” (HFD) (Fig. 6). We hypothesize that MGD corresponds to a tetramer of the HFD of XCAP1 and that each arm domain in the high state (Arm-HS) corresponds to a heterotetramer containing a dimer of the CARP domain of XCAP1 and two G-actin molecules (Fig. 6). The HFD of CAP by itself forms a dimer (12, 26, 27), and the N-terminal oligomerization motif forms a putative coiled-coil and mediates formation of a tetramer (34) or hexamer (10, 24, 25) of the HFD. The height of MGD (Figs. 2*C*, 3*F* and 4*F*) matches with that of the diameter of one HFD (12, 26), suggesting that each HFD is laterally attached to the substrate. By contrast, the C-terminal dimerization motif mediates rigid dimerization of the CARP domain through strand-exchange β-sheet formation (28), which then binds to two G-actin molecules (33). Again, the height of Arm-HS (Fig. 2*E*) matches with the diameter of the heterotetramer of CARP and G-actin (33). Also, the conversion between Arm-LS and Arm-HS is caused by association and dissociation of G-actin (Fig. 5), supporting further that G-actin is a component of Arm-HS. Proline-rich regions and Wiskott Aldrich Syndrome protein-homology 2 (WH2) can serve as a flexible linker between HFD and CARP (Fig. 6). WH2 of CAP binds to G-actin (32, 39), but transient dissociation of WH2 from G-actin can allow full extension of the linker with structural flexibility. WH2 of CAP also binds to the N-terminal diaphanous inhibitory domain of INF2 (23). Therefore, flexibility of WH2 in the CAP–actin complex should keep it accessible with INF2. This hypothetical architecture of the XCAP1–actin complex needs to be tested by additional structural analysis at higher resolutions or localization of components using specific probes.

The configuration of the XCAP1–actin complex in a 4:4 stoichiometric ratio is different from other reported configurations of CAP–actin complexes from different organisms in a 6:6 M ratio (8, 24). It is worth noting that the tripartite structure of the XCAP1–actin complex is very similar to one of the electron microscopy images of yeast Srv2/CAP–actin complex (see Fig. 1 of Ref. (8)). However, the yeast Srv2/CAP–actin complex is a 600-kDa complex containing Srv2/CAP and actin in a molar ratio of 6:6 (8). It remains unclear whether such a difference represents an inherent structural variety of the complex assembly or technical artifacts in the analyses or preparation methods. Further studies are needed to test whether the XCAP1–actin complex with a similar configuration can be reconstituted from purified XCAP1 and G-actin, which should allow dissection of domains and residues that are required for assembly of the XCAP1–actin complex.

Our structural model of the CAP–actin complex places the actin-binding site of HFD outward, suggesting that any two of the four HFDs can interact with two actin subunits at the pointed end of a filament to accelerate depolymerization (12, 13). It allows two remaining free HFD to interact with newly exposed actin subunits at the pointed end, such that CAP can stay bound to a filament and processively depolymerize actin filaments from the pointed end. CAP binds to the pointed end of ADF/cofilin-bound actin filaments with a dwell time of 0.4 to 2.2 s (12, 13), but the precise mechanism of enhanced actin depolymerization remains to be determined. In addition, the CARP domain, which has nucleotide exchange activity, is in proximity and available to capture newly depolymerized ADP-actin and promote rapid conversion to ATP-actin. It would be interesting to determine whether binding of other proteins to WH2 or proline-rich region in the flexible linker (23, 40, 41, 42) or phosphorylation of CAP (43, 44) alters the structure and function of the CAP–actin complex. Thus, our structural model provides mechanistic insight into the function of CAP in the regulation of actin turnover.

## Experimental procedures

### Purification of XCAP1–actin complex from *Xenopus laevis* oocytes

Extracts from *X. laevis* oocytes were prepared and applied to an affinity column in which GST-fused *Xenopus* ADF/cofilin (XAC) had been immobilized as described (35). Proteins bound to the column were eluted with 1 M NaCl, 2 mM MgCl_2_, 1 mM dithiothreitol (DTT), 0.01% NaN_3_, and 20 mM HEPES-KOH, pH 7.2. The eluate was fractionated with ammonium sulfate at 45% saturation. The precipitates obtained by centrifugation were dissolved and dialyzed against 60 mM NaCl buffer (60 mM NaCl, 0.5 mM DTT, 0.01% NaN_3_, and 20 mM Tris-HCl, pH 8.0), applied to a DE52 column pre-equilibrated with the same buffer, and then eluted with a linear gradient of 60 to 300 mM NaCl. The fractions containing XCAP1–actin complex were directly applied to a hydroxyapatite column pre-equilibrated with 60 mM NaCl buffer and washed thoroughly with the same buffer. XCAP1–actin complex was eluted with a linear gradient of 0 to 300 mM potassium phosphate buffer at pH 8.0. Purified XCAP1–actin complex was concentrated by ultrafiltration with Ultracel-30K (Millipore) and dialyzed against 0.1 M KCl, 2 mM MgCl_2_, 1 mM DTT, 0.01% NaN_3_, and 20 mM HEPES-KOH, pH 7.2.

### Size-exclusion chromatography–multiangle light scattering

Samples were analyzed by a Malvern OmniSEC integrated system (Malvern Pananalytical) with an SRT SEC-300 (Sepax) analytical SEC column. Samples were loaded from an autoinjector sample tray that was kept at 20 °C. Phosphate-buffered saline (pH 7.2) containing 0.02% NaN_3_ was used in a mobile phase. Calibration was done using a bovine serum albumin standard. Data from a refractive index, right-angle light scattering (RALS), low-angle light scattering (LALS), viscosimeter, and a UV PDA detector were collected. The resulting chromatograms were analyzed using triple detection (RI, RALS, and viscosimeter) and the dn/dc from sample concentration was used to calculate molecular weight of the peaks as well as hydrodynamic radius. Molecular weights were calculated with Malvern OmniSEC software version 10.41.

### Analytical ultracentrifugation

Sedimentation velocity data were collected using a Beckman Optima AUC analytical ultracentrifuge using a rotor speed of 40,000 rpm (128,794*g*) at 20 °C. Data were recorded by monitoring the sedimentation of the absorbance at 280 nm using a radial step size of 0.001 cm. Set parameters included a partial specific volume (Vbar) of 0.73, a buffer viscosity of 1.002 P, and density of 1.00 g/ml. Sedimentation velocity data were analyzed using both SEDFIT (www.analyticalultracentrifugation.com) (45) and UltraScan (www.ultrascan.uthscsa.edu) (46). Continuous sedimentation coefficient distribution c(*s*) analyses were restrained by maximum entropy regularization at *p* = 0.95 confidence interval. The baseline, meniscus, frictional coefficient, systematic time-invariant and radial-invariant noise were fit.

### Preparation of ADP-G-actin

Actin was prepared as ATP-G-actin from rabbit skeletal muscle as described by Pardee and Spudich (47) and converted to ADP-G-actin as described by Pollard *et al.* (48) by finally dialyzing against buffer containing 0.2 mM ADP, 40 μM MgCl_2_, 0.005% NaN_3_, 0.1 mM DTT, 2 mM Tris-HCl, pH 8.0.

### High-speed atomic force microscopy

HS-AFM imaging was performed in solution at room temperature using a laboratory-built HS-AFM setup (49, 50), as described previously (51). In brief, a glass sample stage (diameter, 2 mm; height, 2 mm) with a thin mica disc (1.5 mm in diameter and ∼0.05 mm in thickness) glued to the top by epoxy was attached onto the top of a Z-scanner by a drop of nail polish. Either bare mica surface or APTES ((3-aminopropyl)triethoxysilane)-treated mica surface (52) was used as a substrate. Onto either substrate, a drop (2 μl) of diluted protein sample (ca. 1 nM) with buffer A (100 mM KCl, 20 mM HEPES–KOH, pH 7.2, 2 mM MgCl_2_) was deposited for 3 min. Then, the surface was rinsed with 20 μl of buffer A and imaged in buffer A. AFM imaging was carried out in a tapping mode using small cantilevers (BLAC10DS-A2, Olympus) (resonant frequency, ∼0.5 MHz in water; quality factor, ∼1.5 in water; spring constant, ∼0.1 N m^−1^). The probe tip was grown on the original tip end of a cantilever through electron beam deposition using ferrocene and was further sharpened using a radio-frequency plasma etcher (Tergeo, PIE Scientific LLC) under an argon gas atmosphere (Direct mode, 10 sccm, and 20 W for 1.5 min). The cantilever’s free oscillation amplitude *A*_0_ and set-point amplitude *A*_s_ were set at ∼2 nm and ∼0.9 × *A*_0_, respectively. The imaging rate, scan size, and pixel size for each AFM image are described in the figure legends.

### Data analyses of HS-AFM images

HS-AFM images were viewed and analyzed using the laboratory built software, Kodec4.4.7.39 (53). In brief, a low-pass filter to remove spike noise and a flattening filter to make the xy-plane flat were applied to individual images. The position and height of the peak within each domain were determined semiautomatically using the following steps. First, the most probable highest point was selected manually. Second, the actual highest point was automatically determined by searching a 5 × 5 pixel area (typically 6.25 × 6.25 nm^2^) around the selected point.

### Molecular graphics

A model of the CAP–actin complex was generated using PyMol (Schrödinger, LLC) and annotated using Adobe Illustrator CS2 (Adobe).

## Data availability

All data are contained in the article. Raw data are available from S. O. upon request.

## Supporting information

This article contains supporting information.

## Conflict of interest

The authors declare no conflict of interest.
