# Supplementary material for: Native cyclase-associated protein and actin from Xenopus laevis oocytes form a unique 4:4 complex with a tripartite structure
Source: J Biol Chem. 2021 Apr 9;296:100649. doi: 10.1016/j.jbc.2021.100649 (PMC8113726; doi:10.1016/j.jbc.2021.100649)
Supplement: Supplementary Materials [file mmc5.pdf]

Supplementary Materials

Supplementary Movie S1. HS-AFM movie showing a typical XCAP1-Actin complex on a mica surface. Representative frames are shown in Fig. 2. Scanning area, 80 × 64 nm^2 with 64 × 48 pixels; imaging rate, 66 ms/frame (~15 fps); z-scale, 0 – 9 nm. Green and blue arrowheads indicate the binding and dissociation events of actin molecules on the CARP domain, respectively.

Supplementary Movie S2. HS-AFM movie showing a typical XCAP1 complex on a mica surface. Representative frames are shown in Fig. 3. Scanning area, 80 × 64 nm^2 with 64 × 48 pixels; imaging rate, 66 ms/frame (~15 fps). Z-scales for upper and lower movies are 0 – 9 nm and 0 – 4.5 nm, respectively.

Supplementary Movie S3. HS-AFM movie showing a typical XCAP1 complex on an APTES treated mica surface. Representative frames are shown in Fig. 4. Scanning area, 100 × 100 nm^2 with 80 × 80 pixels; imaging rate, 100 ms/frame (10 fps); Z-scale, 0 – 5 nm.

Supplementary Movie S4. HS-AFM movie of the XCAP1-actin complex showing frequent and reversible conversions between Arm-LS and Arm-HS. The molecule was observed on a mica surface in buffer A after addition of final 100 nM ADP-G-actin. Scanning area, 150 x 150 nm^2 with 80 x 80 pixels; imaging rate, 200 ms/frame (5 fps); Z-scale, 0 – 9 nm. Pink arrowheads indicate conversion events from Arm-LS to Arm-HS.
